# Adeninium 3-carboxy­anilinium bis­(perchlorate) trihydrate

**DOI:** 10.1107/S1600536809034199

**Published:** 2009-09-05

**Authors:** Lamia Bendjeddou, Aouatef Cherouana, Nasreddine Hadjadj, Slimane Dahaoui, Claude Lecomte

**Affiliations:** a Laboratoire de Chimie Moléculaire, du Contrôle de l’Environnement et des Mesures Physico-Chimiques, Faculté des Sciences Exactes, Département de Chimie, Université Mentouri de Constantine, 25000 Constantine, Algeria; b Cristallographie, Résonance Magnétique et Modélisation (CRM2), Université Henri Poincaré, Nancy 1, Faculté des Sciences, BP 70239, 54506 Vandoeuvre lès Nancy CEDEX, France

## Abstract

In the title salt, C_5_H_6_N_5_^+^·C_7_H_8_NO_2_^+^·2ClO_4_^−^·3H_2_O, the 3-carboxy­anilinium and adeninium cations are monoprotonated at the amino group and at a pyrimidine N atom respectively. In the crystal, the components are involved in extensive three-dimensional hydrogen-bonding networks composed of O—H⋯O, N—H⋯O, O—H⋯N, N—H⋯N and C—H⋯O inter­actions. Bifurcated hydrogen bonds are observed between perchlorate O atoms and adeninium cations.

## Related literature

For hydrogen bonds in hybrid compounds, see: Baker *et al.* (1992 ▶); Richards *et al.* (1972 ▶). Hydrogen-bonding patterns involving amino­pyrimidine and carboxyl­ates have been observed in drug-receptor inter­actions, protein-nucleic acid inter­actions and supra­molecular architectures, see: Perutz & Ten Eyck (1972 ▶). For their applications in drug design and the crystal engineering of pharmaceuticals, see: Desiraju (1989 ▶). For the use of amino­pyrimidine derivatives as anti­folate drugs, see: Stanley *et al.* (2005 ▶); Hunt *et al.* (1980 ▶). For studies of cation–anion hydrogen-bonding in organic salts of carboxylic acids, see: Bendjeddou *et al.* (2003 ▶, 2009 ▶); Cherouana *et al.* (2003 ▶); Moussa Slimane *et al.* (2009 ▶). For the dependence of bond lengths and angles in adeninium cations on the degree of protonation, see: Hingerty *et al.* (1981 ▶); Langer & Huml (1978 ▶). For bond angles in unprotonated adenine, see: Voet & Rich (1970 ▶). For the hydrogen-bonding pattern in adeninium perchlorate adenine dihydrate, see: Zeleňák *et al.* (2004 ▶). For hydrogen-bond motifs, see: Bernstein *et al.* (1995 ▶). For a description of the Cambridge Structural Database, see: Allen *et al.* (1987 ▶). 
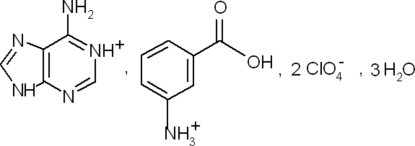


## Experimental

### 

#### Crystal data

C_5_H_6_N_5_^+^·C_7_H_8_NO_2_^+^·2ClO_4_^−^·3H_2_O
*M* *_r_* = 527.24
Triclinic, 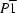

*a* = 8.95610 (10) Å
*b* = 10.5563 (2) Å
*c* = 11.7362 (2) Å
α = 71.431 (7)°
β = 85.800 (5)°
γ = 78.192 (4)°
*V* = 1029.52 (5) Å^3^
*Z* = 2
Mo *K*α radiation
μ = 0.40 mm^−1^
*T* = 120 K
0.16 × 0.1 × 0.08 mm

#### Data collection

Nonius KappaCCD diffractometer
Absorption correction: none
55756 measured reflections
6914 independent reflections
5822 reflections with *I* > 2σ(*I*)
*R*_int_ = 0.028

#### Refinement

*R*[*F* ^2^ > 2σ(*F* ^2^)] = 0.032
*wR*(*F* ^2^) = 0.098
*S* = 0.97
6914 reflections
316 parameters
9 restraints
H atoms treated by a mixture of independent and constrained refinement
Δρ_max_ = 0.70 e Å^−3^
Δρ_min_ = −0.64 e Å^−3^

### 

Data collection: *CAD-4 Software* (Enraf–Nonius, 1989 ▶); cell refinement: *DENZO* and *SCALEPACK* (Otwinowski & Minor, 1997 ▶); data reduction: *DENZO* and *SCALEPACK*; program(s) used to solve structure: *SIR92* (Altomare *et al.*, 1993 ▶); program(s) used to refine structure: *SHELXL97* (Sheldrick, 2008 ▶); molecular graphics: *ORTEP-3* (Farrugia, 1997 ▶); software used to prepare material for publication: *WinGX* (Farrugia, 1999 ▶), *PARST97* (Nardelli, 1995 ▶), *Mercury* (Macrae *et al.*, 2006 ▶) and *POVRay* (Persistence of Vision Team, 2004 ▶).

## Supplementary Material

Crystal structure: contains datablocks global, I. DOI: 10.1107/S1600536809034199/at2870sup1.cif

Structure factors: contains datablocks I. DOI: 10.1107/S1600536809034199/at2870Isup2.hkl

Additional supplementary materials:  crystallographic information; 3D view; checkCIF report

## Figures and Tables

**Table 1:** Hydrogen-bond geometry (Å, °)

*D*—H⋯*A*	*D*—H	H⋯*A*	*D*⋯*A*	*D*—H⋯*A*
N1*A*—H1*A*⋯O2*W*	0.86	2.03	2.8135 (13)	151
N1*A*—H1*A*⋯O6	0.86	2.54	3.0145 (14)	115
O1*M*—H1*M*⋯N7*A*^i^	0.82	1.86	2.6676 (14)	167
N1—H1*N*⋯O1*W*^ii^	0.89	1.86	2.7381 (15)	171
N1—H2*N*⋯O2*W*	0.89	1.92	2.8111 (14)	174
N1—H3*N*⋯O7^iii^	0.89	2.00	2.8539 (14)	162
N9*A*—H9*A*⋯N3*A*^iv^	0.86	2.07	2.9013 (14)	163
O3*W*—H13*W*⋯O1^v^	0.868 (15)	2.297 (14)	3.0378 (18)	143.5 (13)
O3*W*—H13*W*⋯O1*M*^iii^	0.868 (15)	2.548 (16)	3.0127 (14)	114.5 (11)
O1*W*—H21*W*⋯O4^ii^	0.843 (15)	2.149 (13)	2.9329 (14)	154.6 (18)
O1*W*—H21*W*⋯O7^vi^	0.843 (15)	2.49 (2)	3.0553 (14)	124.9 (14)
O2*W*—H22*W*⋯O3	0.841 (14)	2.466 (15)	2.9164 (16)	114.5 (13)
O2*W*—H22*W*⋯O3^ii^	0.841 (14)	2.161 (15)	2.9627 (16)	159.3 (16)
O3*W*—H23*W*⋯O6	0.869 (17)	2.062 (17)	2.9199 (14)	169.5 (16)
N6*A*—H61⋯O1*W*	0.86	2.46	2.9458 (15)	116
N6*A*—H61⋯O8^vi^	0.86	2.33	3.0126 (15)	137
N6*A*—H62⋯O2*M*^vii^	0.86	1.97	2.8187 (14)	167
C2*A*—H2*A*⋯O6	0.93	2.50	3.0047 (15)	115
C2*A*—H2*A*⋯O7^iii^	0.93	2.48	3.2166 (15)	136
C5*M*—H5*M*⋯O1^ii^	0.93	2.52	3.3883 (17)	156
C8*A*—H8*A*⋯O5^viii^	0.93	2.58	3.2881 (15)	133
C8*A*—H8*A*⋯O3*W*^ix^	0.93	2.42	3.1994 (16)	142

Symmetry codes: (i) 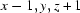; (ii) 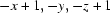; (iii) 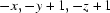; (iv) 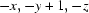; (v) 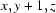; (vi) 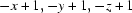; (vii) 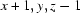; (viii) 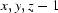; (ix) 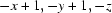.

**Fig. 1.**
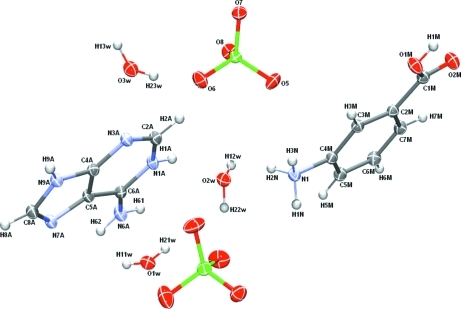
The asymmetric unit of (I), showing the atom-labelling scheme and the hydrogen bonds within the selected asymmetric unit (dashed lines). Displacement ellipsoids are drawn at the 50% probability level and H atoms are shown as small spheres of arbitrary radii.

**Fig. 2.**
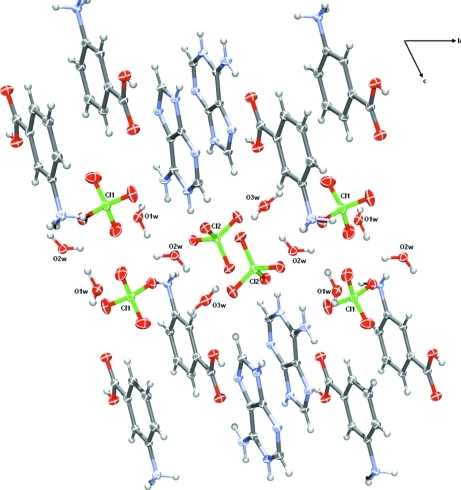
A packing diagram for the title compound, viewed along the a axis, showing the formation of layers.

**Fig. 3.**
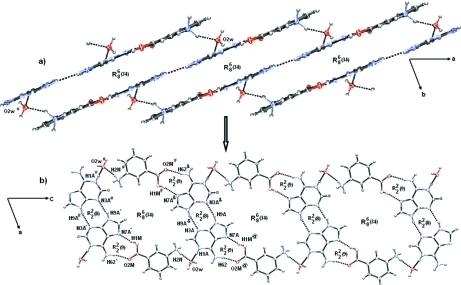
View of the two-dimensional hydrogen-bonded network parallel to the (001) and (101) planes of (I), showing the aggregation of R22(8), R22(9) and R42(34) hydrogen-bonding motifs. Atoms marked with a star (*), a hash symbol (#), an ampersand (&) or an at sign (@), are at the symmetry positions (-1 + x, y, 1 + z), (-1 + x, y, z), (x, 1 - y, z), (1 + x, y - 1 + z), respectively.

**Fig. 4.**
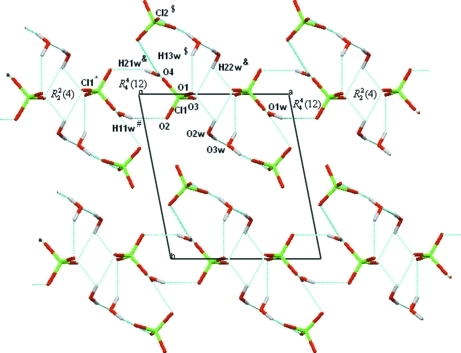
Part of the crystal structure, showing the aggregation of R44(12) and R22(4) motifs via O—H···O hydrogen bonds. Atoms marked with a hash symbol (#), an ampersand (&), dollar sign ($), or a star (*) are at the symmetry positions (-1 + x, y, z), (1 - x, -y, 1 - z), (x, -1 + y, z), (-x, -y, 1 - z), respectively.

**Fig. 5.**
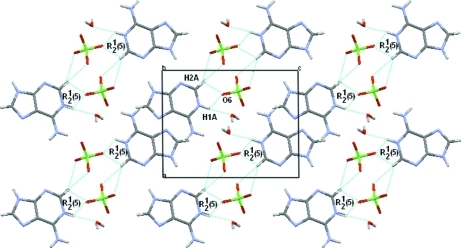
Bifurcate hydrogen bonding

## supplementary crystallographic information

### Comment

Hydrogen bonds of hybrid compounds are of interest because of their widespread biological occurrence (Baker *et al.*, 1992), Richards *et al.*, 1972). Hydrogen-bonding patterns involving aminopyrimidine and carboxylates have been observed in drug-receptor interactions, protein-nucleic acid interactions and supramolecular architectures (Perutz *et al.*, 1972). Studies of such interactions are also of current interest because of their applications in drug design and the crystal engineering of pharmaceuticals (Desiraju *et al.*, 1989). Pyrimidine and aminopyrimidine derivatives are biologically important as they occur in nature as components of nucleic acid. Some aminopyrimidine derivatives are used as antifolate drugs (Stanley *et al.*, 2005; Hunt *et al.*, 1980). The supramolecular networks become especially interesting when the cation and anion can participate in hydrogen-bonding. In this regard previous studies have been concerned with organic salts of carboxylic acids (Bendjeddou *et al.*, 2003; 2009; Cherouana *et al.*, 2003; Moussa Slimane *et al.*, 2009)

Our investigations have focused on the use of perchloric, amino acids and/or nitrogen base acid as a structural building in the synthesis of hydrogen-bonded patterns inorganic-organic high-dimensional structure.

The asymmetric unit of (I) consists of two different monoprotonated adeninium and *m*-carboxyanilinium cations, two perchlorate anions and three water molecules (Fig. 1). A proton transfer from the perchloric acid to atom N1A of the imidazolyl moiety of adenine base and N1 of *m*-carboxyalinine acid resulted in the formation of salts. Adeninium cations can be either mono- or diprotonated and the bond lengths and angles are dependent on the degree of protonation (Hingerty *et al.*, 1981; Langer & Huml, 1978). This form contains three basic N atoms, the most basic site is N1, which accepts the first proton, and the next protonation occurs at N7 and then at N3. In the title compound (I), only atom N1 is protonated. This is evident from the increase in the ring angle at the site of protonation, namely N1. The internal angles at N1 is increased from the reported values of 119.8 in unprotonated adenine (Voet & Rich, 1970). The bond lengths and angles of *m*-carboxyanilinium cation correspond to those expected for the atom types and the type of hybridization (Allen *et al.*, 1987). All bond lengths and angles shows that the two perchlorate anions are tetrahedral.

The title compound is built on the basis of alternating cations and anions chains, the water molecules are sandwiched between them (Fig. 2). In (I), the cationic entities are connected into a two-dimensional hydrogen-bonded network *via* O—H..N, N—H···N and N—H···O hydrogen bonds, thus generating double layers, the junction between them is ensured by a N1A—H1A···O2w and N1—H2N···O2w hydrogen bonds *via* a water molecule (H2O(2)), forming a centrosymmetric rings a long [100] axe which can be described by the graph-set motif of *R*_6_^3^(34) (Bernstein *et al.*, 1995) (Fig. 3a).

The carbonyl O and the carboxyl H atoms participates in hydrogen bonding with a neighbouring adeninium cation through an N—H···O and O—H···N hydrogen bond. The combination of these two hydrogen bonds generates a noncentrosymmetric fused rings which can be described by the graph-set motif of *R*_2_^2^(9). The adeninium cations are linked by two independents N—H···N hydrogen bonds (Table 3), atom N9A (*x*, *y*, *z*) acts as a hydrogen-bond donor to atom N3A at (-*x*, 1 - *y*,-*z*), so generating a Centrosymmetric ring *R*_2_^2^(8). A similar pattern was also observed in the crystal structure of adeninium perchlorate adenine dihydrate (Zeleňák, *et al.*, 2004) (Fig. 3 b).

The water molecules plays a pivotal role, they bridges the perchlorate anions as shown in Fig.4, so forming an alternating of *R*_2_^2^(4) and an *R*_4_^4^(12) rings running parallel to the [100] direction at a = 1/2 & 0 respectively.

The H atoms respectively from protonated atom N1 and atom C2A are involved in bifurcated hydrogen bonding with perchlorate atom O6 to form a five-membered hydrogen-bonded *R*_2_^1^(5) ring into a two-dimensional network (Fig.5).

### Experimental

The compound was obtained as colourless crystals, after few days, by slow evaporation from an aqueous solution of adenine, *m*-carboxyphenyl ammonium and perchloric acid in stoechiometric ratio of 1:1:1.

### Refinement

H atoms were positioned geometrically and refined in the riding-model approximation, with C—H = 0.93 Å, O—H = 0.82 Å, N—H = 0.89 Å and 0.86 Å for ammonium and aromatic H atoms, respectively, with *U*_iso_(H) = 1.2U_eq_(C, N) or 1.5U_eq_(O). The H atoms of the water molecule were located in a difference Fourier map and refined as riding, with O—H = 0.85 Å and *U*_iso_(H) = 1.5U_eq_(O).

### Crystal data

**Table:**

C_5_H_6_N_5_^+^·C_7_H_8_NO_2_^+^·2ClO_4_^−^·3H_2_O	*Z* = 2
*M**_r_* = 527.24	*F*(000) = 544
Triclinic, *P*1	*D*_x_ = 1.701 Mg m^−^^3^
Hall symbol: -P 1	Mo *K*α radiation, λ = 0.71073 Å
*a* = 8.9561 (1) Å	Cell parameters from 55756 reflections
*b* = 10.5563 (2) Å	θ = 1.0–31.6°
*c* = 11.7362 (2) Å	µ = 0.40 mm^−^^1^
α = 71.431 (7)°	*T* = 120 K
β = 85.800 (5)°	Needle, brown
γ = 78.192 (4)°	0.16 × 0.1 × 0.08 mm
*V* = 1029.52 (5) Å^3^	

### Data collection

**Table:**

Nonius KappaCCD diffractometer	5822 reflections with *I* > 2σ(*I*)
Radiation source: fine-focus sealed tube	*R*_int_ = 0.028
graphite	θ_max_ = 31.6°, θ_min_ = 2.8°
ω scans	*h* = 0→13
55756 measured reflections	*k* = −14→15
6914 independent reflections	*l* = −17→17

### Refinement

**Table:**

Refinement on *F*^2^	9 restraints
Least-squares matrix: full	H atoms treated by a mixture of independent and constrained refinement
*R*[*F*^2^ > 2σ(*F*^2^)] = 0.032	*w* = 1/[σ^2^(*F*_o_^2^) + (0.0626*P*)^2^ + 0.328*P*] where *P* = (*F*_o_^2^ + 2*F*_c_^2^)/3
*wR*(*F*^2^) = 0.098	(Δ/σ)_max_ = 0.001
*S* = 0.97	Δρ_max_ = 0.70 e Å^−^^3^
6914 reflections	Δρ_min_ = −0.64 e Å^−^^3^
316 parameters	

### Special details

**Table:**

Geometry. All e.s.d.'s (except the e.s.d. in the dihedral angle between two l.s. planes) are estimated using the full covariance matrix. The cell e.s.d.'s are taken into account individually in the estimation of e.s.d.'s in distances, angles and torsion angles; correlations between e.s.d.'s in cell parameters are only used when they are defined by crystal symmetry. An approximate (isotropic) treatment of cell e.s.d.'s is used for estimating e.s.d.'s involving l.s. planes.

### Fractional atomic coordinates and isotropic or equivalent isotropic displacement parameters (Å^2^)

**Table:**

	*x*	*y*	*z*	*U*_iso_*/*U*_eq_	
Cl2	0.19523 (3)	0.56122 (3)	0.55586 (2)	0.01535 (6)	
Cl1	0.26532 (3)	0.01925 (3)	0.32829 (3)	0.01919 (7)	
O4	0.18456 (11)	−0.09092 (10)	0.38018 (9)	0.02546 (19)	
O8	0.32466 (10)	0.59734 (10)	0.59496 (9)	0.02321 (18)	
O2	0.15769 (12)	0.14560 (10)	0.28846 (11)	0.0331 (2)	
O5	0.16150 (11)	0.43814 (9)	0.64068 (9)	0.0262 (2)	
O6	0.22752 (11)	0.54327 (10)	0.43911 (8)	0.0256 (2)	
O1W	0.84304 (11)	0.11571 (10)	0.36340 (9)	0.02322 (18)	
H11W	0.9191 (16)	0.1428 (19)	0.3222 (14)	0.035*	
H21W	0.848 (2)	0.1307 (19)	0.4293 (11)	0.035*	
O2M	−0.17505 (10)	0.23783 (10)	1.07286 (8)	0.02331 (18)	
O3	0.36504 (13)	0.02378 (12)	0.41750 (10)	0.0337 (2)	
O3W	0.46761 (11)	0.68985 (11)	0.31943 (9)	0.02467 (19)	
H23W	0.4054 (18)	0.6368 (16)	0.3575 (16)	0.037*	
H13W	0.4085 (18)	0.7627 (12)	0.2763 (15)	0.037*	
O2W	0.41376 (10)	0.23763 (9)	0.51016 (8)	0.01987 (17)	
H22W	0.4707 (17)	0.1627 (12)	0.5132 (16)	0.03*	
H12W	0.4495 (19)	0.2689 (16)	0.5578 (14)	0.03*	
O7	0.06388 (10)	0.66963 (9)	0.54746 (8)	0.02176 (18)	
O1M	−0.27521 (10)	0.26982 (10)	0.89398 (8)	0.02123 (18)	
H1M	−0.3543	0.2954	0.9269	0.032*	
O1	0.35415 (12)	−0.00416 (13)	0.22739 (10)	0.0358 (3)	
N3A	0.11767 (11)	0.45851 (10)	0.13693 (9)	0.01576 (17)	
N9A	0.19753 (11)	0.44133 (10)	−0.06160 (8)	0.01525 (17)	
H9A	0.1122	0.4736	−0.0989	0.018*	
N1A	0.32637 (11)	0.37745 (10)	0.27036 (8)	0.01595 (18)	
H1A	0.355	0.3641	0.3423	0.019*	
N7A	0.44549 (11)	0.35292 (10)	−0.02915 (9)	0.01645 (18)	
N1	0.15132 (11)	0.14796 (10)	0.62650 (9)	0.01761 (18)	
H3N	0.0719	0.2065	0.5864	0.026*	
H1N	0.1521	0.0656	0.6211	0.026*	
H2N	0.2373	0.1751	0.5951	0.026*	
N6A	0.57570 (11)	0.29041 (11)	0.22322 (9)	0.0204 (2)	
H61	0.6025	0.2766	0.2957	0.024*	
H62	0.642	0.2695	0.1721	0.024*	
C4A	0.22236 (12)	0.42565 (11)	0.05563 (10)	0.01409 (19)	
C2M	−0.01192 (12)	0.18475 (11)	0.91804 (10)	0.01548 (19)	
C6M	0.25407 (13)	0.08172 (13)	0.94549 (11)	0.0204 (2)	
H6M	0.3389	0.0453	0.9949	0.025*	
C5M	0.26698 (13)	0.08683 (12)	0.82521 (11)	0.0194 (2)	
H5M	0.3596	0.0535	0.7939	0.023*	
C5A	0.37585 (12)	0.37129 (11)	0.07482 (10)	0.01494 (19)	
C1M	−0.16129 (13)	0.23298 (12)	0.96985 (10)	0.0169 (2)	
C4M	0.13915 (13)	0.14247 (11)	0.75291 (10)	0.01572 (19)	
C6A	0.43380 (13)	0.34339 (11)	0.19055 (10)	0.0158 (2)	
C3M	−0.00021 (13)	0.19153 (11)	0.79698 (10)	0.01574 (19)	
H3M	−0.0846	0.2284	0.7471	0.019*	
C2A	0.17701 (13)	0.43122 (12)	0.24266 (10)	0.0168 (2)	
H2A	0.1123	0.45	0.3034	0.02*	
C7M	0.11526 (13)	0.13073 (12)	0.99212 (11)	0.0182 (2)	
H7M	0.1073	0.1275	1.0724	0.022*	
C8A	0.33410 (13)	0.39600 (12)	−0.10839 (10)	0.0164 (2)	
H8A	0.3475	0.3954	−0.1876	0.02*	

### Atomic displacement parameters (Å^2^)

**Table:**

	*U*^11^	*U*^22^	*U*^33^	*U*^12^	*U*^13^	*U*^23^
Cl2	0.01578 (12)	0.01495 (12)	0.01556 (12)	−0.00305 (9)	−0.00132 (8)	−0.00477 (9)
Cl1	0.01813 (12)	0.01781 (13)	0.02080 (13)	−0.00179 (9)	−0.00226 (9)	−0.00546 (10)
O4	0.0315 (5)	0.0199 (4)	0.0263 (5)	−0.0088 (4)	−0.0028 (4)	−0.0060 (4)
O8	0.0197 (4)	0.0283 (5)	0.0253 (4)	−0.0077 (3)	−0.0044 (3)	−0.0105 (4)
O2	0.0240 (5)	0.0175 (4)	0.0522 (7)	0.0008 (4)	−0.0025 (4)	−0.0057 (4)
O5	0.0276 (5)	0.0169 (4)	0.0291 (5)	−0.0063 (3)	−0.0021 (4)	0.0013 (3)
O6	0.0291 (5)	0.0330 (5)	0.0210 (4)	−0.0087 (4)	0.0028 (4)	−0.0161 (4)
O1W	0.0230 (4)	0.0241 (4)	0.0248 (4)	−0.0031 (3)	−0.0008 (3)	−0.0114 (4)
O2M	0.0197 (4)	0.0324 (5)	0.0181 (4)	−0.0018 (4)	0.0001 (3)	−0.0103 (4)
O3	0.0338 (5)	0.0363 (6)	0.0382 (6)	−0.0078 (4)	−0.0126 (4)	−0.0182 (5)
O3W	0.0210 (4)	0.0292 (5)	0.0232 (4)	−0.0015 (4)	−0.0013 (3)	−0.0093 (4)
O2W	0.0203 (4)	0.0218 (4)	0.0183 (4)	−0.0027 (3)	−0.0025 (3)	−0.0077 (3)
O7	0.0185 (4)	0.0191 (4)	0.0241 (4)	0.0018 (3)	−0.0016 (3)	−0.0047 (3)
O1M	0.0134 (4)	0.0311 (5)	0.0186 (4)	0.0004 (3)	−0.0002 (3)	−0.0100 (4)
O1	0.0268 (5)	0.0494 (7)	0.0241 (5)	0.0021 (5)	0.0032 (4)	−0.0080 (5)
N3A	0.0147 (4)	0.0177 (4)	0.0152 (4)	−0.0025 (3)	0.0004 (3)	−0.0061 (3)
N9A	0.0131 (4)	0.0185 (4)	0.0136 (4)	−0.0010 (3)	−0.0019 (3)	−0.0051 (3)
N1A	0.0154 (4)	0.0217 (5)	0.0120 (4)	−0.0044 (3)	−0.0003 (3)	−0.0065 (3)
N7A	0.0143 (4)	0.0204 (4)	0.0146 (4)	−0.0018 (3)	0.0006 (3)	−0.0066 (3)
N1	0.0159 (4)	0.0186 (4)	0.0170 (4)	−0.0014 (3)	0.0008 (3)	−0.0051 (4)
N6A	0.0153 (4)	0.0295 (5)	0.0152 (4)	−0.0007 (4)	−0.0030 (3)	−0.0069 (4)
C4A	0.0145 (4)	0.0140 (4)	0.0136 (4)	−0.0024 (4)	−0.0011 (4)	−0.0041 (4)
C2M	0.0147 (5)	0.0150 (5)	0.0164 (5)	−0.0032 (4)	0.0001 (4)	−0.0044 (4)
C6M	0.0160 (5)	0.0218 (5)	0.0207 (5)	−0.0010 (4)	−0.0038 (4)	−0.0036 (4)
C5M	0.0149 (5)	0.0196 (5)	0.0213 (5)	−0.0011 (4)	−0.0001 (4)	−0.0044 (4)
C5A	0.0139 (4)	0.0170 (5)	0.0141 (5)	−0.0027 (4)	−0.0008 (4)	−0.0051 (4)
C1M	0.0162 (5)	0.0166 (5)	0.0176 (5)	−0.0028 (4)	−0.0005 (4)	−0.0047 (4)
C4M	0.0154 (5)	0.0152 (5)	0.0158 (5)	−0.0030 (4)	0.0002 (4)	−0.0038 (4)
C6A	0.0161 (5)	0.0167 (5)	0.0149 (5)	−0.0038 (4)	0.0004 (4)	−0.0050 (4)
C3M	0.0141 (4)	0.0151 (5)	0.0174 (5)	−0.0021 (4)	−0.0009 (4)	−0.0044 (4)
C2A	0.0152 (5)	0.0194 (5)	0.0162 (5)	−0.0034 (4)	0.0005 (4)	−0.0064 (4)
C7M	0.0176 (5)	0.0187 (5)	0.0171 (5)	−0.0032 (4)	−0.0021 (4)	−0.0038 (4)
C8A	0.0161 (5)	0.0188 (5)	0.0143 (5)	−0.0023 (4)	0.0002 (4)	−0.0059 (4)

### Geometric parameters (Å, °)

**Table:**

Cl2—O5	1.4363 (9)	N7A—C8A	1.3220 (14)
Cl2—O8	1.4387 (9)	N7A—C5A	1.3804 (14)
Cl2—O6	1.4429 (9)	N1—C4M	1.4630 (15)
Cl2—O7	1.4483 (9)	N1—H3N	0.89
Cl1—O1	1.4367 (11)	N1—H1N	0.89
Cl1—O2	1.4386 (10)	N1—H2N	0.89
Cl1—O4	1.4405 (10)	N6A—C6A	1.3110 (15)
Cl1—O3	1.4432 (10)	N6A—H61	0.86
O1W—H11W	0.851 (9)	N6A—H62	0.86
O1W—H21W	0.843 (9)	C4A—C5A	1.3819 (15)
O2M—C1M	1.2225 (14)	C2M—C7M	1.3936 (16)
O3W—H23W	0.869 (9)	C2M—C3M	1.3969 (16)
O3W—H13W	0.868 (9)	C2M—C1M	1.4893 (16)
O2W—H22W	0.840 (9)	C6M—C7M	1.3894 (17)
O2W—H12W	0.843 (9)	C6M—C5M	1.3925 (17)
O1M—C1M	1.3173 (14)	C6M—H6M	0.93
O1M—H1M	0.82	C5M—C4M	1.3876 (16)
N3A—C2A	1.3082 (14)	C5M—H5M	0.93
N3A—C4A	1.3625 (14)	C5A—C6A	1.4096 (15)
N9A—C4A	1.3618 (14)	C4M—C3M	1.3802 (15)
N9A—C8A	1.3623 (14)	C3M—H3M	0.93
N9A—H9A	0.86	C2A—H2A	0.93
N1A—C2A	1.3612 (14)	C7M—H7M	0.93
N1A—C6A	1.3687 (14)	C8A—H8A	0.93
N1A—H1A	0.86		
			
O5—Cl2—O8	110.25 (6)	N3A—C4A—C5A	127.04 (10)
O5—Cl2—O6	110.09 (6)	C7M—C2M—C3M	120.44 (10)
O8—Cl2—O6	109.38 (6)	C7M—C2M—C1M	119.27 (10)
O5—Cl2—O7	108.72 (6)	C3M—C2M—C1M	120.28 (10)
O8—Cl2—O7	109.21 (6)	C7M—C6M—C5M	120.43 (11)
O6—Cl2—O7	109.17 (6)	C7M—C6M—H6M	119.8
O1—Cl1—O2	109.41 (7)	C5M—C6M—H6M	119.8
O1—Cl1—O4	108.98 (7)	C4M—C5M—C6M	118.80 (11)
O2—Cl1—O4	109.54 (6)	C4M—C5M—H5M	120.6
O1—Cl1—O3	109.60 (7)	C6M—C5M—H5M	120.6
O2—Cl1—O3	110.26 (7)	C4A—C5A—N7A	110.32 (9)
O4—Cl1—O3	109.03 (6)	C4A—C5A—C6A	118.20 (10)
H11W—O1W—H21W	105.5 (15)	N7A—C5A—C6A	131.47 (10)
H23W—O3W—H13W	104.3 (14)	O2M—C1M—O1M	123.91 (11)
H22W—O2W—H12W	107.2 (14)	O2M—C1M—C2M	122.61 (11)
C1M—O1M—H1M	109.5	O1M—C1M—C2M	113.47 (10)
C2A—N3A—C4A	112.44 (10)	C3M—C4M—C5M	121.97 (11)
C4A—N9A—C8A	106.73 (9)	C3M—C4M—N1	118.77 (10)
C4A—N9A—H9A	126.6	C5M—C4M—N1	119.25 (10)
C8A—N9A—H9A	126.6	N6A—C6A—N1A	120.99 (10)
C2A—N1A—C6A	123.92 (10)	N6A—C6A—C5A	125.47 (10)
C2A—N1A—H1A	118	N1A—C6A—C5A	113.54 (10)
C6A—N1A—H1A	118	C4M—C3M—C2M	118.65 (10)
C8A—N7A—C5A	104.25 (9)	C4M—C3M—H3M	120.7
C4M—N1—H3N	109.5	C2M—C3M—H3M	120.7
C4M—N1—H1N	109.5	N3A—C2A—N1A	124.84 (10)
H3N—N1—H1N	109.5	N3A—C2A—H2A	117.6
C4M—N1—H2N	109.5	N1A—C2A—H2A	117.6
H3N—N1—H2N	109.5	C6M—C7M—C2M	119.70 (11)
H1N—N1—H2N	109.5	C6M—C7M—H7M	120.2
C6A—N6A—H61	120	C2M—C7M—H7M	120.2
C6A—N6A—H62	120	N7A—C8A—N9A	112.76 (10)
H61—N6A—H62	120	N7A—C8A—H8A	123.6
N9A—C4A—N3A	127.02 (10)	N9A—C8A—H8A	123.6
N9A—C4A—C5A	105.94 (9)		

### Hydrogen-bond geometry (Å, °)

**Table:**

*D*—H···*A*	*D*—H	H···*A*	*D*···*A*	*D*—H···*A*
N1A—H1A···O2W	0.86	2.03	2.8135 (13)	151
N1A—H1A···O6	0.86	2.54	3.0145 (14)	115
O1M—H1M···N7A^i^	0.82	1.86	2.6676 (14)	167
N1—H1N···O1W^ii^	0.89	1.86	2.7381 (15)	171
N1—H2N···O2W	0.89	1.92	2.8111 (14)	174
N1—H3N···O7^iii^	0.89	2.00	2.8539 (14)	162
N9A—H9A···N3A^iv^	0.86	2.07	2.9013 (14)	163
O3W—H13W···O1^v^	0.87 (2)	2.30 (1)	3.0378 (18)	144 (1)
O3W—H13W···O1M^iii^	0.87 (2)	2.55 (2)	3.0127 (14)	115 (1)
O1W—H21W···O4^ii^	0.84 (2)	2.15 (1)	2.9329 (14)	155 (2)
O1W—H21W···O7^vi^	0.84 (2)	2.49 (2)	3.0553 (14)	125 (1)
O2W—H22W···O3	0.84 (1)	2.47 (2)	2.9164 (16)	115 (1)
O2W—H22W···O3^ii^	0.84 (1)	2.16 (2)	2.9627 (16)	159 (2)
O3W—H23W···O6	0.87 (2)	2.06 (2)	2.9199 (14)	170 (2)
N6A—H61···O1W	0.86	2.46	2.9458 (15)	116
N6A—H61···O8^vi^	0.86	2.33	3.0126 (15)	137
N6A—H62···O2M^vii^	0.86	1.97	2.8187 (14)	167
C2A—H2A···O6	0.93	2.50	3.0047 (15)	115
C2A—H2A···O7^iii^	0.93	2.48	3.2166 (15)	136
C5M—H5M···O1^ii^	0.93	2.52	3.3883 (17)	156
C8A—H8A···O5^viii^	0.93	2.58	3.2881 (15)	133
C8A—H8A···O3W^ix^	0.93	2.42	3.1994 (16)	142

Symmetry codes: (i) *x*−1, *y*, *z*+1; (ii) −*x*+1, −*y*, −*z*+1; (iii) −*x*, −*y*+1, −*z*+1; (iv) −*x*, −*y*+1, −*z*; (v) *x*, *y*+1, *z*; (vi) −*x*+1, −*y*+1, −*z*+1; (vii) *x*+1, *y*, *z*−1; (viii) *x*, *y*, *z*−1; (ix) −*x*+1, −*y*+1, −*z*.
